# Colonization–persistence trade-offs in natural bacterial communities

**DOI:** 10.1098/rspb.2023.0709

**Published:** 2023-07-12

**Authors:** Vicente J. Ontiveros, José A. Capitán, Emilio O. Casamayor, David Alonso

**Affiliations:** ^1^ Theoretical and Computational Ecology, Center for Advanced Studies of Blanes (CEAB-CSIC), Spanish Council for Scientific Research, Accés Cala St. Francesc 14, E-17300 Blanes, Spain; ^2^ Integrative Freshwater Ecology Group, Centre of Advanced Studies of Blanes (CEAB-CSIC), Spanish Council for Scientific Research, Accés Cala St. Francesc 14, E-17300 Blanes, Spain; ^3^ Complex Systems Group. Department of Applied Mathematics, Universidad Politécnica de Madrid. Av. Juan de Herrera, 6. E-28040 Madrid, Spain

**Keywords:** colonization–extinction dynamics, species coexistence, natural bacterial communities, species sorting, neutral theory, fitness equalization

## Abstract

Fitness equalizing mechanisms, such as trade-offs, are recognized as one of the main factors promoting species coexistence in community ecology. However, they have rarely been explored in microbial communities. Although microbial communities are highly diverse, the coexistence of their multiple taxa is largely attributed to niche differences and high dispersal rates, following the principle ‘everything is everywhere, but the environment selects’. We use a dynamical stochastic model based on the theory of island biogeography to study highly diverse bacterial communities over time across three different systems (soils, alpine lakes and shallow saline lakes). Assuming fitness equalization mechanisms, here we newly analytically derive colonization–persistence trade-offs, and report a signal of such trade-offs in natural bacterial communities. Moreover, we show that different subsets of species in the community drive this trade-off. Rare taxa, which are occasional and more likely to follow independent colonization/extinction dynamics, drive this trade-off in the aquatic communities, while the core sub-community did it in the soils. We conclude that equalizing mechanisms may be more important than previously recognized in bacterial communities. Our work also emphasizes the fundamental value of dynamical models for understanding temporal patterns and processes in highly diverse communities.

## 1. Introduction

Understanding the coexistence of a high number of species is a long recurring theme in ecology [1]. Contemporary coexistence theory indicates that there are two major classes of mechanisms that promote coexistence: *stabilizing* mechanisms that increase negative frequency-dependent selection, and *equalizing* mechanisms that reduce fitness differences among species [2]. Stabilizing mechanisms comprise resource partitioning, disease or storage effects [3], while equalizing mechanisms such as trade-offs are generally produced by life-history trait evolution in a context of historical contingency [4]. Although many examples of trade-offs can be found in macroscopic communities (e.g. [5–7]), few have been shown for microbes, usually in experimental metacommunities (e.g. [8,9]). To the best of our knowledge, equalizing mechanisms in natural microbial communities have not been carefully evaluated yet. In this paper, our goal is to examine the role of a colonization–persistence trade-off in promoting coexistence across natural bacterial communities in both terrestrial and aquatic ecosystems.

Despite the key insight of Chesson [2], ecologists are unable to predict species coexistence in an open area [10]. Metacommunity ecology [11,12] tries to understand species coexistence and biodiversity, recognizing the importance of scale and spatio-temporal processes. Currently, metacommunity ecology is characterized by four distinct archetypes: species sorting (SS), which focuses on how local environmental conditions enable some species to coexist; neutral theory (NT), which centres on dispersal limitation and demographic stochasticity; patch dynamics (PD), which concentrates on the balance of colonization and extinction processes in relatively homogeneous patches; and mass effects (ME), which emphasizes that dispersal may outweigh competitive forces in a set of heterogeneous patches. These archetypes can be associated with coexistence mechanisms. Adler *et al.* [13] relate SS and NT with stabilizing and equalizing mechanisms, respectively. SS is related to niche differences, while in NT, dispersal limitation and stochasticity associated with demographic processes override fitness differences resulting in equalization. In the PD archetype, species diversity is maintained by equalizing mechanisms, such as trade-offs in colonization and competitive ability [14–16], or survival/fecundity and competition [17,18].

The advent of next-generation sequencing (NGS) techniques has shown that the microbiota is highly diverse. Traditionally, microbial diversity has been explained by the principle ‘everything is everywhere, but the environment selects’ [19,20]. Thus, microbial diversity is usually understood by appealing to the formation of highly interacting microbial associations maintained by niche differences, emphasizing that stabilizing mechanisms underlie microbial coexistence. This interpretation of the principle neglects the effects of dispersal [19,21,22], a potential equalizing mechanism whose importance may be substantial in some communities [23]. Until recent times, microbial ecology has made few mentions of coexistence-promoting mechanisms when analysing microbial communities, with some noteworthy exceptions [9,23–25]. However, microbial ecologists have recently started to talk in terms of general theoretical frameworks in ecology, such as community assembly and metacommunity ecology [22,26]. In fact, there has been considerable debate on whether SS or NT dominates as an assembly mechanism in microbial communities, with a somewhat inconclusive result [27–29] favouring the SS paradigm [30].

Interestingly, it has been conjectured that the relative importance of assembly mechanisms might differ for distinct components of the microbial communities [31–33]. Along similar lines, Hanski [34] already proposed the *core–satellite hypothesis*, framed within the PD archetype, where stochastic variation in colonization and extinction rates leads to species falling into two distinct categories: core species (abundant and persistent) and satellite species (occasional and rare). Magurran & Henderson [35] extended the relevance of the core–satellite hypothesis into the temporal domain, finding that core species display a species abundance distribution compatible with a lognormal distribution, while satellite species follow a log-series. These differences were associated with distinct functional roles for these two components. Microbial ecologists have also identified core and satellite species [36]. Thus, the maintenance of species coexistence in highly dynamic communities, such as the microbial ones, should not be constrained to a single dominant mechanism.

**Figure 1.**
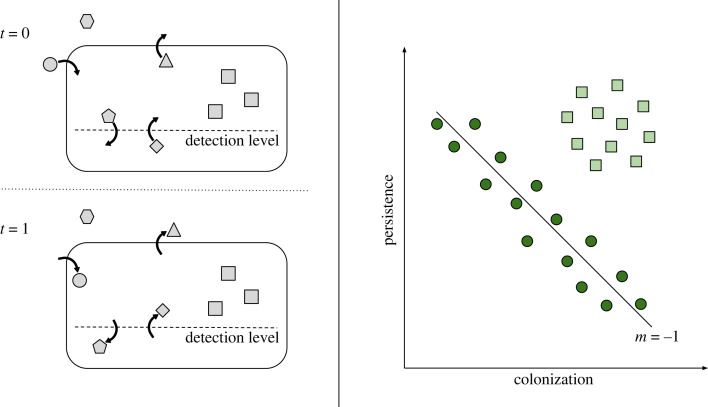
Conceptual summary of this article. The left panel shows a schematic view of colonization-extinction dynamics in microbial communities. In a local community, species can colonize (circle), get extinct (triangle), or maintain their presence (squares). However, detection levels can produce taxa to get apparently extinct (pentagon) or apparently colonize (diamond), which can be a confounding factor. Still, colonization–extinction events allow for the estimation of colonization and persistence in a consistent way. In the right panel, fitness equalization produces a relationship between colonization and persistence with a slope of −1 in logarithmic space. The solid line indicates a perfect persistence–colonization trade-off, where equalizing mechanisms such as trade-offs lead to similar fitness among groups. Any attempt of the satellite taxa (dark circles) to increase their performance would likely result in a corresponding decrease due to life-history constraints. However, in core taxa (light squares) stabilizing mechanisms dominate and niche differences are high (e.g. due to resource partitioning).

Here, we investigate the role of equalizing coexistence mechanisms in bacterial communities. Our initial hypothesis is that natural bacterial communities, which are species-rich, should show a signal of fitness equalization. However, we hypothesize that the different components of the community might be affected by contrasting mechanisms and the satellite sub-community will be more prone to display signals of fitness equalization (figure 1). In this article, we first derive analytically a colonization–persistence trade-off from the hypothesis of fitness equalization in the context of colonization–extinction dynamics. Then, we validate our estimates of colonization and extinction rates in two replicated, longitudinal studies of bacterial communities. After that, using species presence–absence temporal data, we report a signal of the colonization-persistence trade-off characterizing three metacommunities coherently at different taxonomical levels. Finally, we found that the relative influence of coexistence promoting mechanisms is different for core and satellite taxa, although not always the satellite component is closer to the theoretical expectation of fitness equalization. Recognizing the importance of equalizing mechanisms may render a better understanding of the functioning of highly diverse microbial communities.

## 2. Material and methods

### (a) Data samples

We have selected three temporal samplings of free-living, natural bacterial communities with some degree of replication to perform our study. First, we used temporal samples from the water column of four high-altitude lakes in the Spanish Pyrenees, monthly followed during 1 year [37,38]. Of the four lakes, three of them were connected by the same stream, while the remaining one is located in another basin. Second, we examined soil samples from two sites in Switzerland, after a soil compaction experiment, followed for 4 years [39]. This longitudinal sampling presents the rare feature of containing replicate samples, which combined with the three treatments in the experiments, four different temporal samples and two sites, produced 3 × 3 × 4 × 2 = 72 samples of the soil community. Third, we used data from 12 shallow saline lakes in the Spanish Monegros desert plateau, monthly sampled along 3 years and covering different dry–wet periods [40], encompassing a total of 122 different samples of this aquatic metacommunity. Further details can be found in the original publications. Bacterial communities were studied after NGS of DNA 16S rRNA amplicon analyses, denoising and chimera removal, then clustered at 97% OTU identity, and transformed to presence–absence data. Sequence processing and genetic data analyses were carried out as reported in the original studies, except for the Pyrenees dataset, where we used the UPARSE pipeline [41]. Additional ecological and environmental information can be found in the original publications. The complete genetic datasets are available in GenBank under BioProject record IDs PRJNA566370 (Pyrenean lakes), PRJNA429605 (Monegros) and as supplemental material for the Swiss soils [39]. Processed data can be found in Dryad [42].

### (b) Colonization and extinction rates

Throughout this work, we applied the simplest stochastic model underlying TIB [43–45]. This dynamic model explains the average level of richness and its variation in a study site (or *island*) in terms of colonization and extinction processes, on the one hand, and the total number of potentially colonizing species in the regional pool, or metacommunity richness, on the other hand. As Hanski [46] showed, this model can be derived from an ensemble of single-species models of presence–absence dynamics, under the assumptions of both species independence and equivalence [43]. So, we can estimate the model parameters for the dynamics of the whole community from presence–absence temporal data, and, therefore, we characterize the entire bacterial community by a single colonization-extinction pair. Alternatively, we can subdivide the community in guilds, relaxing the equivalence assumption, and estimate a distinct and characteristic colonization-extinction pair for each of them [43,47]. To calculate colonization (*c*) and extinction (*e*) rates from the observed presences and absences, as the bacterial communities were sampled following an *irregular sampling scheme* (samples separated by unequal time intervals), we used the functions irregular_single_dataset and irregular_multiple_datasets from R package ‘island’ [47]. Note that for microbial taxa, these colonization and extinction rates are *effective*, as local events can be affected by imperfect detectability. Here, we consider that a *colonization* has taken place in a time interval Δ*t* when a detection failure (whether the taxon is present or not) at time *t* follows a detection success at time *t* + Δ*t*. Conversely, a taxon *goes extinct* in a time interval Δ*t* when it was successfully detected at time *t*, but was under the detection level a time *t* + Δ*t* (whether present or not; see figure 1). Thus we are implying that taxa below detection levels are few and, therefore, relatively unimportant for microbial community patterns and dynamics. However, for the Swiss soils, we also used methods that account for imperfect detectability [47] to estimate the colonization and extinction rates, as replicate samplings were available. A detailed account of these methods and the results obtained with them can be found in the electronic supplementary material.

As a way to assess the applicability of the method to bacterial communities, we started estimating colonization-extinction rates for several independent sites. For the Pyrenees dataset, we compared three lakes from the same basin (Lakes Llebreta, Llong and Redó d’Aigüestortes [37]) and one in a different basin (Lake Redó [38]). We followed a model selection procedure, based on the Akaike information criterion and the weight of evidence *w*_*i*_ [48], to develop a series of models with different sets of partitions of the four lakes, and estimate, for each of these partitions, a pair of colonization and extinction rates. Besides, we used data from the Swiss soils to test the precision of the method when confronted with replicates of the same community. Once we assessed the correct performance of the method, we subdivided whole communities into different taxonomic levels, which we considered as ecologically equivalent guilds, for the three habitats under study. Note that the estimation of colonization-extinction rates for very labile taxa might be biased. Therefore, we excluded, from subsequent analyses, those taxa with an estimated persistence value, defined as the inverse of the extinction rate (*p*_*i*_ ≡ 1/*e*_*i*_), much shorter than the minimal inter-event sampling time (less than approximately a quarter of this time).

### (c) Core and satellite members of the community

Multiple methods have been applied to distinguish between core and satellite members of a community. While core species are abundant and persistent, satellite species usually show up at lower abundances and are occasional or even accidental. These two components of ecological communities feature distinct functional characteristics. The fact that persistent members of the community usually follow a lognormal abundance distribution, while accidental species follow the log-series [35], can be potentially used as a method to sort out the community core from the rest. However, when processing sequence data from microbial samples, it is common practice to discard OTU sequences appearing only once to minimize potential errors. Therefore, the log-series distribution is difficult or impossible to assess since it requires to record all real singleton species possibly observed in the sample. Instead of using abundance distributions directly, we have applied Chow tests to identify structural breaks in the relation between logarithmic maximum abundances and occupancy (defined as the probability that a species appears in the community over time). The Chow test [49] aims to identify unexpected changes in the parameters of linear regression models along the range of the independent variable. We first identified the intermediate breakpoint with the highest Chow test’s statistic, this leading to two different slopes in the abundance–occupancy relation. Then we estimated the mean occupancy between consecutive ends of the two regression lines. We defined as core members of the community those OTUs with occupancy values higher than the aforementioned mean occupancy. OTUs with occupancy values below this threshold were identified as satellite members of the community. We performed Chow tests using the R package ‘strucchange’ [50] and lognormal fits for the core sub-community using the R package ‘vegan’ [51].

## 3. Results

### (a) A colonization–persistence trade-off

Trade-offs in ecology arise due to multiple mechanisms, such as competition, perturbations or physiological constraints. Trade-offs tend to equalize fitness across species. In the context of colonization–extinction models, the colonization to extinction ratio can be regarded as a good measure of species fitness [15]. In fact, it represents the number of new colonization events during the average time a species remains present in the system before extinction. If two species share this number, they should reach the same importance in the system, either measured in terms of average abundance or average presence. This is true whether species follow Levin’s metapopulation dynamics [15], or simple colonization-extinction independent dynamics, as we used in this paper. Under the assumption of species dynamics independence, species metacommunity dynamics can be formulated as

3.1 $$\frac{d\pi_{i}}{dt}=c_{i}(1-\pi_{i})-e_{i}\pi_{i},$$

where (*c*_*i*_, *e*_*i*_) stands for the colonization–extinction rate pair for species *i* belonging to a pool of size *S*_*P*_ (*i* = 1, 2, …, *S*_*P*_), and *π*_*i*_ is the probability that species *i* is found in a community (i.e. the occupancy of that species). Therefore, the probability of species *i* being present at equilibrium can be written as

3.2 $$\pi_{i}^{⋆}=\frac{k_{i}}{1+k_{i}},$$

where *k*_*i*_ = *c*_*i*_/*e*_*i*_ is the colonization to extinction ratio. Now we assume that equalizing mechanisms drive community dynamics, hence we expect that species fitness tends to equalize among species, $k_{1}\approx k_{2}\approx \cdots \approx k_{S_{p}}$. Therefore, the probability $\pi_{i}^{⋆}$, which is also called expected occupancy at stationarity, tends to equalize for those species that share the same dimensionless colonization to extinction ratio, *k*. Conversely, if steady-state occupancies $\pi_{i}^{⋆}$ are assumed to be roughly equal across species, then equation (3.2) trivially implies that all colonization to extinction ratios (*k*_*i*_) will tend to be constant across species. Henceforth, we defined persistence as the inverse of the extinction rate (*p*_*i*_ ≡ 1/*e*_*i*_). Because the hypothesis of equalizing mechanisms implies that all ratios *c*_*i*_/*e*_*i*_ are approximately constant, *c*_*i*_/*e*_*i*_ = *c*_*i*_ *p*_*i*_ ≈ *k*, we find the following persistence–colonization fitness-equalizing trade-off:

3.3 $$p_{i}=k\;c_{i}^{-1}.$$

A generic colonization–persistence trade-off can be conceptualized as

3.4 $$p_{i}=k\;c_{i}^{\alpha },$$

with exponent *α* < 0. Therefore, we conclude that if a community of equivalent species is close to performing colonization-extinction independent dynamics, the exponent *α* of the generic colonization–persistence trade-off above (equation (3.4)) should be −1. This is our theoretical prediction, the one we have checked across the three different bacterial communities. Throughout this work, we have represented colonization and persistence axes on a logarithmic scale. This leads us to conclude that a trade-off between colonization and persistence compatible with independent colonization-extinction dynamics should display a slope equal to −1 on a log scale, as it is deduced from equation (3.3): log*p*_*i*_ = *K* − log*c*_*i*_, where *K* = log *k*.

### (b) The species equivalence assumption

Under this assumption, all species in the community are described by the same colonization-extinction pair. This approximation allowed us to explore whole community dynamics for bacteria in lakes of the Pyrenees and soils in Switzerland. For the lakes in the Pyrenees (electronic supplementary material, figure S1A), we found that the dynamics of the three lakes in the same basin were so similar that they accumulated a weight of evidence of 89% (summing over all models considering at least two of the three lakes as having the same colonization and extinction), as opposed to the model with all lakes with different rates, which had only a weight of evidence of 11% (see table S1 in electronic supplementary material). In the case of the soils in Switzerland (electronic supplementary material, figure S1B), the distance among replicas within the same soil type and site in colonization and extinction rates was smaller than between sites, showing that the replicas had similar dynamics on each site.

### (c) Relaxing the equivalence assumption

**Figure 2.**
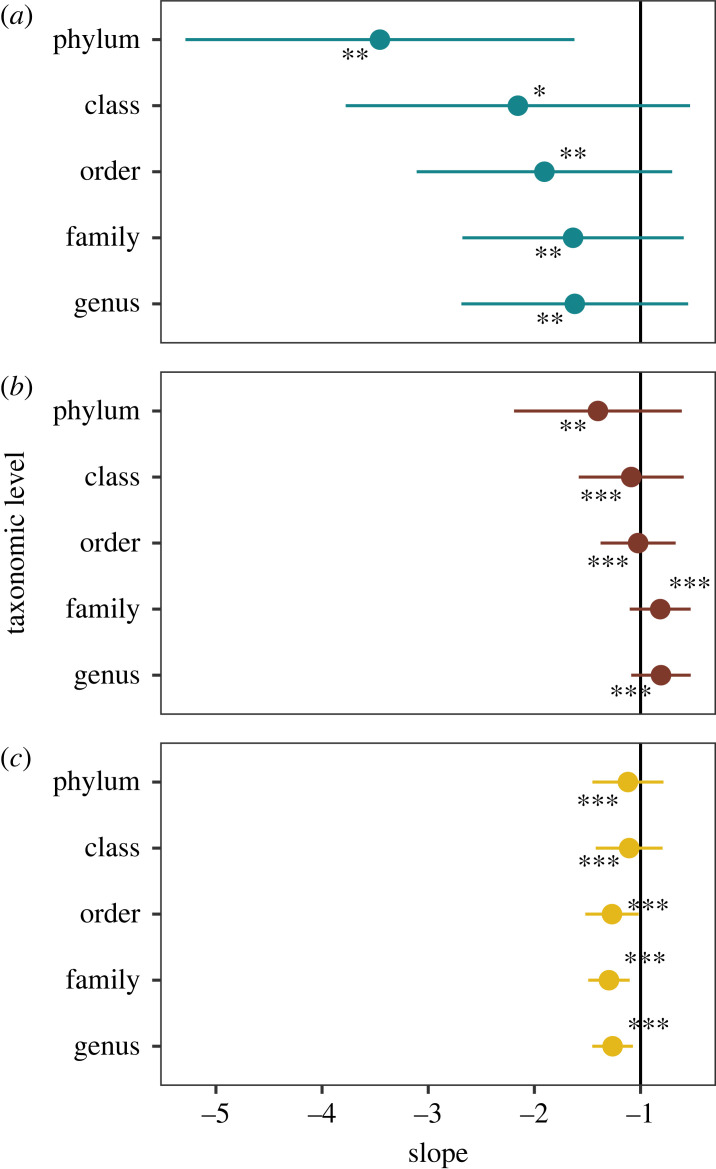
The three communities studied display a signal of fitness equalization. (*a*) Lakes in the Pyrenees, (*b*) soils and (*c*) shallow saline lakes in Monegros. The slope of the relationship of colonization and persistence was close to −1 along the different taxonomic levels, indicating fitness equalization. Significance refers to Spearman’s *ρ*, * <0.05, ** <0.01, *** <0.001.

Next, we relaxed this assumption and considered the different taxonomic groups in these two bacterial communities, plus the metacommunity in saline lagoons in the Monegros desert. We found that colonization-persistence patterns were coherent as we descended to lower taxonomic levels. So, the distances of genera and families within phyla, classes, or orders (intra-group) were lower than distances between different higher taxonomic levels (inter-group) in the three communities (Kruskal–Wallis test, all *p*-values less than 0.1 in the Pyrenees and less than 0.01 in saline lagoons and soils). Moreover, our estimates of colonization and persistence were negatively related conforming to a generic trade-off. An increase in colonization rates led to decreases in persistence, and this relation was maintained across taxonomic levels for the three communities (figure 2, electronic supplementary material, table S2). Also, the slope of the linear models relating the logarithms of colonization and persistence was very close to −1. We recall here that a slope of −1 would correspond to a colonization-persistence trade-off resulting from fitness equalization between different taxonomic groups, under the assumption of colonization–extinction independent dynamics.

However, the assumptions underlying a colonization–persistence trade-off with exponent −1 might be too severe to apply to whole communities. It is well-known that core members of a community may display different dynamics from the satellite components of it [35]. Satellite species tend to be rare and accidental. Sometimes they are observed, sometimes they are not. These species may be good candidates to show a kind of behaviour consistent with colonization–extinction independent dynamics, and, therefore, the satellite subcommunity should tend to show, accordingly, a colonization–persistence trade-off with exponent −1. Instead, the core members of the community tend to be more abundant, and therefore the relative strength of niche processes, such as interactions and niche segregation, would be higher than in the case of satellite species. Then, core species would not necessarily show a trade-off with exponent −1 if they show any at all.

**Figure 3.**
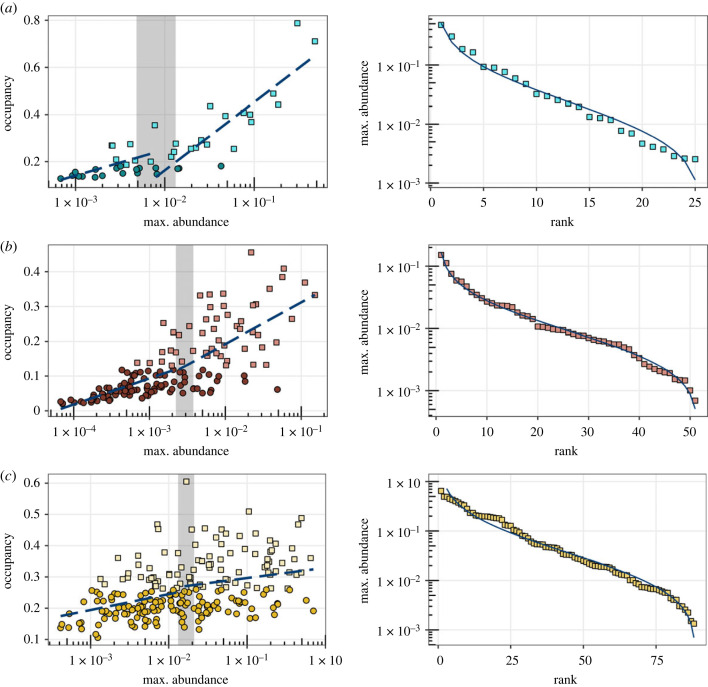
The core members of the community follow a lognormal distribution. (*a*) Lakes in the Pyrenees, (*b*) soils and (*c*) shallow saline lakes in Monegros. Left panels: blue dashed lines represent the linear relationship between the highest abundance and occupancy at the genus level, which presents structural changes, determined by a Chow test with maximum values for the statistic in the grey shaded area. We have considered as core genera (squares) those that presented values of occupancy higher than the mean occupancy of the point with maximum structural change, while those with a lesser occupancy were considered satellite members (circles). Right: the core members of the communities present a lognormal distribution (solid blue line). Pyrenees deviance = 1.063; soils deviance = 0.666; Monegros deviance = 4.042. Lognormal distributions were fitted using function rad.lognormal of the R package ‘vegan’.

To test this hypothesis, we first identified the core and the satellite members of our communities. As abundance enhances occupancy, following a similar argument as in Magurran & Henderson [35], we represented the linear relation among maximum abundance and occupancy at the genus level. However, we used a Chow test analysis instead to separate the core from the satellite members of the community by identifying structural changes in the linear relation among maximum abundance and occupancy. Figure 3 shows the structural changes found in the three studied communities. The point with the biggest statistic allowed us to infer an occupancy threshold that separated the core from the satellite members of the community. As reported for macroscopic communities, the abundance distribution of the core sub-communities followed a lognormal distribution closely.

**Figure 4.**
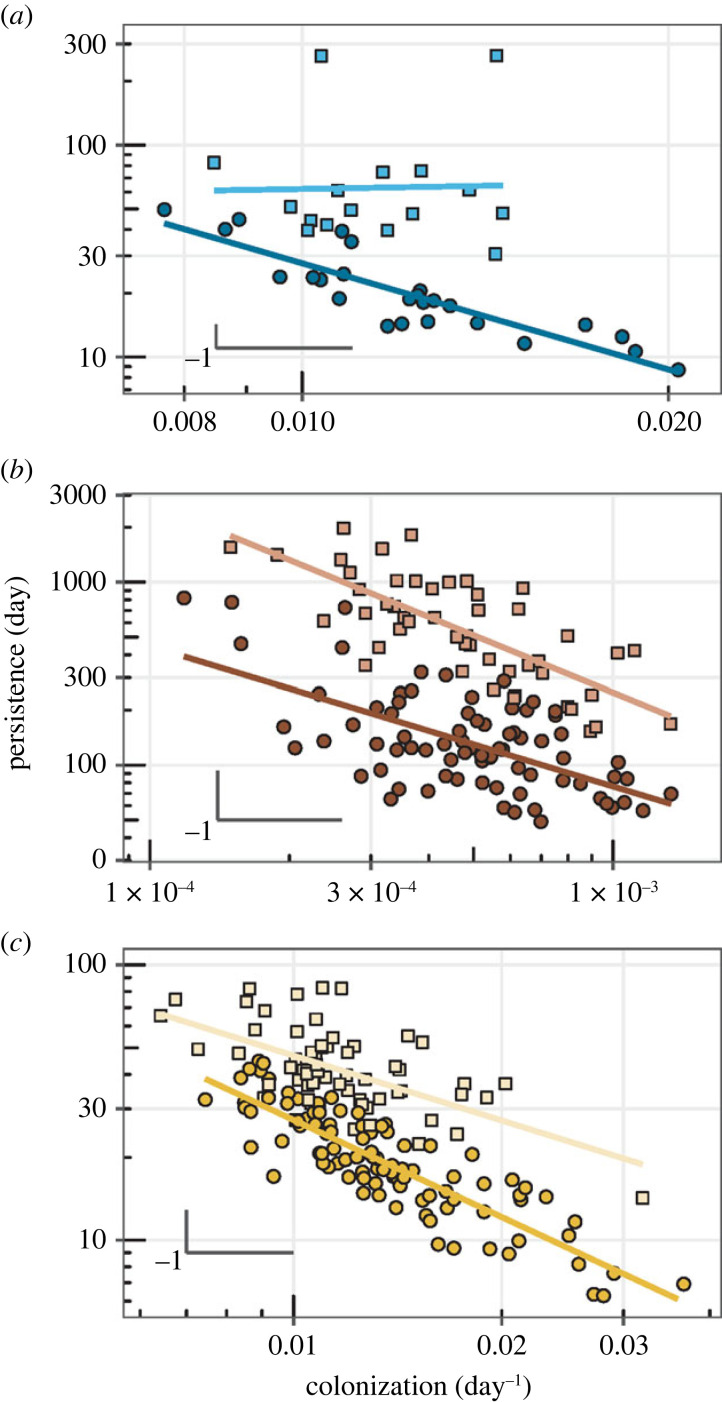
Bacterial communities show a colonization–persistence trade-off at the family level. Three different habitats—(*a*) alpine lakes, (*b*) soils and (*c*) shallow saline lakes—display a linear relationship close to the theoretical expectation under a perfect colonization-persistence trade-off (not shown). The trade-off is maintained throughout the phylogeny, from phylum to genus. However, core (squares) and satellite (circles) members of the community show different relationships between persistence and colonization, being the satellite members closer to the theoretical expectation. The two legs indicate the −1 slope.

In sum, the distinction between core and satellite members of the community allowed us to examine the relationship between colonization and persistence separately for these two components, as shown at the family taxonomic level (figure 4). These two components presented significantly different slopes, as shown by testing the hypothesis that satellite and core species share the same slope of the linear model (in logarithmic axes) relating colonization and persistence. Moreover, the satellite component of the communities showed slopes very close to −1, while slopes were lower for the community core, except in the case of soils, where both core and satellite sub-communities showed exponents close to −1 but the satellite slope was higher than that of the core (table 1). These results were maintained across taxonomic levels, down to the lowest level, that of genus. However, as we go up in the taxonomy, losses in statistical power blur these relationships.

**Table 1.** Core and satellite sub-communities show differential relationships for colonization and persistence. A slope of −1 would correspond to a perfect trade-off between colonization and persistence. We have tested the hypothesis that the slope of the linear model for satellite taxa is equal to the slope obtained for core species (Student’s *t*-test), which was rejected in all cases. Associated *p*-values and *t*-scores are shown. Additionally, we report data for fitted slopes and their 95% confidence interval.

community	tax.	*p-*value	*t*-score	component	slope	lower C.I.	upper C.I.	*n*
Pyrenees	family	8 × 10^−5^	−5.513	core	−0.168 ^n.s.^	−1.651	1.315	25
				satellite	−1.149 ^***^	−1.531	−0.767	16
	genera	3 × 10^−5^	−5.913	core	−0.103 ^n.s.^	−1.637	1.430	25
				satellite	−1.148 ^***^	−1.527	−0.769	16
soils	family	0.0012	3.366	core	−1.038 ^***^	−1.314	−0.762	49
				satellite	−0.688 ^***^	−0.895	−0.482	78
	genera	0.0004	3.697	core	−1.056 ^***^	−1.319	−0.792	51
				satellite	−0.683 ^***^	−0.883	−0.482	85
Monegros	family	3 × 10^−7^	−5.528	core	−0.784 ^***^	−1.054	−0.514	63
				satellite	−1.172 ^***^	−1.311	−1.033	97
	genera	2 × 10^−5^	−4.428	core	−0.861 ^***^	−1.112	−0.610	88
				satellite	−1.132 ^***^	−1.253	−1.011	112

^n.s.^*p*-value higher than 0.1.

^***^p-value lower than 0.001.

## 4. Discussion

In this study, we have shown analytically that fitness equalization in communities under colonization–extinction dynamics adopts the shape of a colonization-persistence trade-off, with slope −1 in logarithmic space. When this pattern is empirically confronted to data, the trade-off signal was present in the three studied bacterial meta-communities. Moreover, different components of the community displayed a contrasting pattern of their colonization–persistence relationship. However, contrary to our expectation that the satellite sub-community would drive the trade-off, the core sub-community does so in the soil community we studied here. Many taxa would remain in the metacommunity by either evolving higher colonization rates but persisting shorter periods or developing the ability to stay longer in the community along with lower colonization rates. Therefore, this result highlights the importance of fitness equalizing mechanisms in microbial communities. Examples of life-history trade-offs can be found easily among macroorganisms, experimental settings of microbes [52], and even host-associated microbes [23], but, to the best of our knowledge, this is the first time that such a trade-off is reported in highly diverse free-living microbial meta-communities.

We expected bacterial communities to show a relationship between persistence and colonization such as the one conceptualized in figure 1. This pattern is reminiscent of the one reported by Cadotte *et al.* [8]. However, here we identify core and satellite taxa and we provide a quantitative expectation for fitness equalization, testable for any kind of community that undergoes colonization-extinction dynamics. The identification of core and satellite species is not new in microbial ecology [36], although similar terms have arisen to refer to the less abundant component, such as the rare biosphere [53], or conditionally rare taxa [54,55]. We hypothesized that satellite taxa would follow the trade-off as a result of fitness equalizing mechanisms, and, by contrast, core taxa would be driven by stabilizing mechanisms tending to maintain higher persistence than for satellite taxa. Moreover, core taxa are common, abundant species following a lognormal abundance distribution [35]. As Cadotte *et al.* [8] pointed out, in principle, other kinds of taxa could potentially exist: Hutchinsonian ‘demons’, that would competitively exclude other taxa, and evolutionary ‘losers’, that would not colonize nor persist in the community. However, the bacterial communities we have analysed do not fully comply with this conceptual view. Although the three studied communities showed a lognormal abundance distribution for the core component, as expected, and our observation of ecological coherence in colonization and persistence within taxonomic levels might well indicate niche differences [56] produced by stabilizing mechanisms, we found that the core sub-community was driving the trade-off in the studied soils. Still, we found that the core and satellite sub-communities display a contrasting pattern in the three samplings, as could be expected [31].

Several factors might help us understand the departure of the soil community from our expectation. First, we could consider the experiment performed in the soil community as a confounding factor. While the bacterial aquatic communities might be considered at a seasonally driven steady state, the soil community was intentionally poised out from its natural steady state due to a compaction experiment. For instance, soil compaction might have led to increased anaerobiosis driving the community out of and far away from a previous natural colonization-extinction equilibrium. Furthermore, the relaxation time to the new steady state in response to this disturbance might have also differed for the different treatments [39]. However, when we examined the effect of the different treatments on the colonization-persistence pattern, we did not find qualitative differences between them (see electronic supplementary material), so we discarded this explanation. A second factor to take into account is the phylogenetic history of the soil community. Although different patterns of diversification have been associated with fitness or niche differences [25], the diversification pattern of the three studied communities was similar (electronic supplementary material, figure S2). However, it has also been shown that fitness differences are lower for species evolved in sympatry than in allopatry [57]. While soil communities can be considered as mostly sympatric, aquatic communities have an inflow of members of other compartments such as soils or sediments [58,59] and, so, some degree of allopatric components. Therefore, the core component in soils might present similar fitness because of its sympatric evolution. Third, core and satellite taxa might be adscribed to different life-history strategies, e.g. copio- and oligotrophic strategies [60], in soil and aquatic communities. We examined if the core and satellite subcommunities were associated with copio- or oligotrophic lifestyles, and while in the soil community we did not find this association, in the hypersaline community we found that the core subcommunity was deprived of oligotrophic taxa (more details in electronic supplementary material). Still, further research is needed to relate different life-history strategies to coexistence mechanisms. Finally, other factors point to fitness equalization in soil communities, such as a high niche overlap [61], physico-chemical conditions allowing for multiple trade-offs [62], or high rates of horizontal gene transfer (HGT) [63,64]. Therefore, fitness-equalizing mechanisms in the soil core sub-community may be possible.

In the context of metacommunities, stabilizing forces have been associated with SS, while equalizing forces to NT [13]. SS and NT have been proposed alternatively as the major mechanisms controlling microbial community assembly. In fact, the importance of SS has been evaluated against other metacommunity archetypes as NT [27,31] or mass effects (ME) [28,65] with contrasting results. Also, NT has been tested and proposed as the dominant force structuring communities [29,66]. The dichotomies niche–neutral [67,68] or stochastic–deterministic [69–71] are similar to the SS–NT divide, and are often used as synonyms. The most accepted view seems to be that initial steps in community assembly are dominated by neutral processes, while SS characterizes later stages, but this view is rarely put in the context of coexistence mechanisms. Our work adds to this discussion the fact that different components of the communities can be governed by fitness equalization. In the light of our findings, acknowledging the possibility of equalizing mechanisms structuring microbial communities would provide a more comprehensive view of microbial diversity.

Equalizing mechanisms can evolve in species-rich communities with strong dispersal and recruitment limitation [72], although microbial communities are unlikely affected by these limitations. However, experimental settings have repeatedly shown that microbial trade-offs evolve easily in controlled, species-poor experiments [73,74], and might be key in microbial communities [23,75]. A potential equalizing mechanism might be HGT, as it has been proposed that it produces highly flexible gene pools associated with specific habitats [76], that would equalize fitness and increase niche overlap. However, HGT is still difficult to measure *in situ* [77]. Also, nonlinear responses to fluctuating environments can act as equalizing or stabilizing mechanisms [2]. Stabilizing mechanisms are widespread in microbial communities (e.g. resource partitioning, dormancy [78], or cross-feeding [79]), although the processes underlying these mechanisms are rarely studied or understood at trait or biochemical levels. The strength of these stabilizing mechanisms may well allow the satellite members of the community to coexist in the presence of the core component.

Some limitations may affect our study. First, we only use abundance data to distinguish core and satellite taxa. Although the information that taxa abundances provide has been really useful [80,81], modelling efforts in highly diverse microbial communities are still limited. Here, we contribute with a quantitative prediction for fitness equalization that may complement our knowledge on coexistence mechanisms. Second, our results might have been produced by statistical artefacts related to species abundance distributions (SADs). To assess this possibility, we set up simulations that show that SADs alone do not produce a signal of fitness equivalence unless when we enforce it (see electronic supplementary material). Third, our results rely on a dynamic stochastic model, rooted in classic ecological theory. Although its assumptions are drastically simplifying (*species equivalence* and *species independence*), it should be viewed as an approximation to the actual underlying dynamics of the community or its components (when relaxing the *equivalence* assumption). We used this model to estimate extinction and colonization rates from temporal datasets [43,47].

The accuracy of our estimates should be assessed carefully. Firstly, very rare species may be there, but under detectability levels [47,82,83]. Imperfect detectability can only be assessed if enough replicates per sampling time are available. We could only apply these more sophisticated methods to the Swiss soil dataset, as replicate samplings existed. We found similar results to the ones that assumed perfect detectability (electronic supplementary material, tables S3–S4), with a clear relationship between colonization and persistence that, however, showed slightly less pronounced slopes (electronic supplementary material, figure S3). More studies of natural microbial communities with replicated samplings are needed to validate our findings. Secondly, when persistence times are too short compared to inter-sampling times, these estimates may not be reliable [84]. If taxa go in and out from the system too rapidly, their estimated rates may be biased. This possible bias is the reason why we removed some labile taxa (less than 13 % in all cases) from our analyses. The exclusion of these taxa did not change the overall patterns reported in this study. Thirdly, sequence clusterization (e.g. 97% identity versus amplicon sequence variants) may have some influence on our estimates. So, we reanalysed the Monegros dataset following the computational analyses to identify amplicon sequence variants in [85], obtaining equivalent results at the different taxonomic levels we studied and steeper slopes for the core and satellite sub-communities which overlapped their confidence intervals with our original estimates (more details in the electronic supplementary material, tables S5 and S6). Finally, the detection of dead DNA or spores in our samples may bias our estimates. While we anticipate that it will not have a substantial impact on the trade-off (see electronic supplementary material), using methods that account for active cells, such as RNA amplicon, might shed light on this. We have some indication that this DNA just introduces noise in our estimates. When we excluded classes that harbour species known to form the bacterial spore bank (*Actinobacteria*, *Bacilli*, *Clostridia* and other *Firmicutes*, *Deltaproteobacteria* and *Cyanobacteria*) from our analyses, colonization and persistence increased their correlation from 0.637 to 0.7348 (*p*-value = 3 × 10^−10^) in the Monegros dataset at the class level.

The relevance of equalizing mechanisms for coexistence might have been overlooked in natural microbial communities. However, these mechanisms may be relevant in highly diverse ecosystems, acting on different compartments of microbial communities. Here, we unveil a clear signal of fitness equalization through a colonization–persistence trade-off, a pattern that may well be found beyond the microbial world, which would be worth exploring in the future. Long-term temporal studies are needed to improve our knowledge of coexistence mechanisms, along with a better understanding of the influence of microbial life-history strategies, HGT and phylogenetic history over microbial coexistence. We hope that framing this discussion in the context of equalizing versus stabilizing mechanisms would add clarity to current knowledge on the forces maintaining high microbial diversity on Earth ecosystems.

## Data Availability

The complete genetic datasets are publicly available in GenBank under BioProject record IDs PRJNA566370 (Pyrenean lakes), PRJNA429605 (Monegros) and as electronic supplementary material for the Swiss soils (Hartmann *et al.* 2014 [39]). All data and code necessary to reproduce this manuscript is available in Dryad (https://doi.org/10.5061/dryad.gmsbcc2t7) [86] and Zenodo (https://doi.org/10.5281/zenodo.7998019) [87]. Additional information is provided in electronic supplementary material [88].
